# Is Bipolar Radiofrequency Thermotherapy a Reliable Treatment for Benign Prostatic Hyperplasia in Elderly Patients Ineligible for General and Spinal Anesthesia?

**DOI:** 10.7759/cureus.95362

**Published:** 2025-10-24

**Authors:** Ferec Efendioglu, Cihat Özcan, Sanan Asgarli, Ozgur Cinar, Selcuk Sarikaya, Burak Ünal, Selahattin Bedir

**Affiliations:** 1 Department of Urology, Gülhane Training and Research Hospital, Ankara, TUR; 2 Department of Urology, Health Sciences University, Gülhane Training and Research Hospital, Ankara, TUR

**Keywords:** benign prostatic obstruction, bipolar radiofrequency thermotherapy, elderly patients​, lower urinary tract symptoms, minimally invasive therapy

## Abstract

Background

The objective of this study was to investigate the success and safety of bipolar radiofrequency thermotherapy in the treatment of benign prostatic hyperplasia (BPH) in elderly patients ineligible for general and spinal anesthesia owing to significant comorbidities.

Methods

A total of 115 patients were included in the study. The cohort consisted of patients who had BPH refractory to medical treatment along with excessive comorbidities, rendering them unsuitable for general and spinal anesthesia. Patients with preoperative diagnoses of prostate cancer, urethral stricture, median lobe, neurogenic bladder, or psychological disorders were excluded from the study. Prostate-specific antigen (PSA), International Prostate Symptom Score (IPSS), maximum urinary flow rate (Qmax), post-void residual (PVR), and quality of life (QoL) were reassessed, and prostate volume was measured at postoperative months 3 and 12. Preoperative values were compared with values measured at postoperative months 3 and 12. Perioperative complications were assessed according to the Clavien-Dindo Scoring System.

Results

The median age of the patients was 73.71 ± 11.23 years (mean ± SD). Comparison of the preoperative values of prostate volume, PSA, IPSS, Qmax, PVR, and QoL with values measured at postoperative months 3 and 12 showed a significant reduction in prostate volume (p=0.001), significant improvement in IPSS (p=0.001), a significant increase in Qmax (p=0.001), a significant decrease in PVR (p=0.001), and significant improvement in QoL (p=0.01). However, no significant change was noted in PSA values (p=0.976). The VAS score was 4 ± 1 (0-10) at postoperative hour 1. In the early postoperative period (30 days), urinary retention was observed in seven patients (6.08%), and urinary tract infection in four patients (3.4%). Clavien grade 4 or grade 5 complications were not observed.

Conclusions

Bipolar radiofrequency thermotherapy is a successful and safe method for the treatment of BPH in elderly patients who are ineligible for spinal or general anesthesia due to excessive comorbidities.

## Introduction

Lower urinary tract symptoms (LUTS) due to aging are common in older men [1,2]. One factor that causes LUTS is benign prostatic obstruction (BPO) that becomes more prevalent with aging [3,4]. The first-line treatment of BPO typically involves medical treatments such as alpha-1 blockers, 5-alpha reductase inhibitors, etc. [5]. Approximately 36% of the patients under active follow-up for BPO or those who were started on medical treatment for BPO require surgical intervention within five years [6,7]. For patients with prostate volumes less than 80 ml, transurethral resection of the prostate (TURP) is considered the gold standard surgical method [8]. Although TURP is a reliable method, it is associated with complications such as transurethral resection (TUR) syndrome, bleeding, urethral stricture, and retrograde ejaculation. Additionally, the success of TURP is significantly reduced in prostates with volumes more than 80 ml, and it cannot be performed in patients receiving anticoagulants [9,10]. To minimize complications and provide a surgical treatment for BPO in patients with significant comorbidities, a more minimally invasive method, that is, transurethral bipolar radiofrequency prostate ablation (TEMPRO), was developed [11]. The primary objective of TEMPRO is to achieve permanent thermoablation of obstructive prostate tissue while avoiding damage to surrounding structures such as the urethra, rectum, and bladder during the thermoablation process [12]. A review of the relevant published articles revealed that there has been limited research on the effectiveness and safety of the TEMPRO technique.

In this present study, the objective was to investigate the effectiveness and safety of TEMPRO. Furthermore, it was also aimed to ascertain the extent to which TEMPRO reduces preoperative prostate-specific antigen (PSA) levels and prostate volume.

## Materials and methods

The study was conducted at Gülhane Training and Research Hospital between January 2019 and August 2023. Data was collected retrospectively.

The total number of patients was 115. The study included patients who had BPO refractory to medical treatment along with excessive comorbidities (American Society of Anesthesiologists (ASA) score 3 and 4), rendering them unsuitable for general and spinal anesthesia. Patients with a preoperative diagnosis of prostate cancer, urethral stricture, or median lobe enlargement greater than 10 mm on transrectal ultrasound (TRUS) causing bladder neck obstruction were excluded from the study. Those with neurogenic bladder, previous prostate or bladder outlet surgery, or active urinary tract infection were also not eligible. Additionally, individuals diagnosed with psychiatric disorders, including generalized anxiety disorder and major depressive disorder, were excluded to avoid bias in pain and anxiety assessments. Finally, patients who were unable or unwilling to provide informed consent were not enrolled in the study.

Preoperative PSA values, International Prostate Symptom Scores (IPSS), maximum urinary flow rate (Qmax), postvoid residual volume (PVR), quality of life (QoL), and prostate weight were noted. In the early postoperative period (at hour 1), pain assessment was conducted using the Visual Analogue Scale (VAS).

PSA, IPSS, Qmax, PVR, and QoL were reassessed, and prostate weight was measured at postoperative months 3 and 12. Preoperative values were compared with values measured at postoperative months 3 and 12. In addition, preoperative and postoperative data were compared between patients with prostate volumes less than 80 mL and those with volumes greater than or equal to 80 mL.

Perioperative complications were evaluated and classified according to the Clavien-Dindo classification system [13], an open-access and standardized tool for reporting surgical complications.

Preoperatively, all patients were administered third-generation cephalosporins for prophylaxis. Urethral lidocaine gel (2%) was used as a local anesthetic 10 minutes before the insertion of the catheter. In addition, before using the catheter, intramuscular paracetamol (10 mg/1mL/100ml) plus intramuscular diclofenac sodium (75 mg/3mL) were administered (if the creatinine value was normal).

The Direx TEMPRO system and a silicone-coated 16-French (16F) latex catheter with six-ring electrodes were utilized. The Direx TEMPRO system uses a computer-controlled mechanism that directs bipolar radiofrequency (RF) energy to the prostate through feedback from three temperature sensors. Throughout the procedure, the system continuously monitors the intraprostatic temperature, maintaining it at approximately 55 °C without active cooling. Prior to the insertion of the catheter, intra-urethral lidocaine gel (2%) was administered locally. TEMPRO was performed at 55.0 °C for 1 hour, and no cooling process was performed. Thanks to the use of bipolar RF, the heat was concentrated on a small cylinder next to the urethra, meaning that a rectal probe was not needed, pending the operation.

Data analysis was conducted using IBM SPSS Statistics Standard Concurrent User Version 26 (IBM Corp., Armonk, NY, USA). Descriptive statistics were reported in terms of numbers (n), percentage (%), mean ± standard deviation, and minimum-maximum values. For parametric tests, the precondition of variance homogeneity was verified with the Levene test. The normality assumption was tested using the Shapiro-Wilk test. When evaluating differences between the two groups, Student’s t-test was employed if the parametric test prerequisites were met, whereas the Mann-Whitney U test was used otherwise. Mixed Design ANOVA was used in comparisons across different measurement time points. In Mixed Design ANOVA, the Bonferroni correction was employed when comparing the main effects. P<0.05 was considered statistically significant.

The study was approved by the Institutional Review Board of Gülhane Training and Research Hospital (Approval No: 2022-248; Date: January 17, 2023). All participants provided written informed consent.

## Results

A total of 115 patients were included in the study. The mean age was 73.71 ± 11.23 years (mean ± SD). Preoperative prostate volume was 57.68 ± 29.41 ml, Qmax 12.05 ± 1.25 ml/s, PVR 137.56 ± 104.48 ml, and IPSS 19.54 ± 6.16 (all mean ± SD). Thirty-six patients required catheterization prior to surgery (Table 1).

**Table 1:** Demographic characteristics of 115 patients. n: Number of patients; %: column percentage. Numerical variables were reported as mean ± standard deviation and minimum–maximum values. Qmax: Maximum flow rate of urination; PVR: Post-void residual volume; IPSS: International Prostate Symptom Score; QoL: Quality of Life.

Variable	Mean ± SD	Range (min–max)
Age (years)	73.71 ± 11.23	47–96
Preoperative Qmax (ml/s)	12.05 ± 1.25	2.1–13.52
Preoperative PVR (ml)	137.56 ± 104.48	0–500
Preoperative IPSS	19.54 ± 6.16	9–32
Preoperative prostate volume (cc)	57.68 ± 29.41	13–154
Preoperative PSA (ng/ml)	7.55 ± 2.68	0.19–26.5
QoL	4.55 ± 1.17	3–6

The average operative time was 66.21 ± 1.65 minutes. The mean ASA score was 3 ± 1. Seventy-two patients (62.6%) had two systemic comorbidities (such as hypertension, ischemic heart disease, diabetes mellitus, and chronic obstructive pulmonary disease), while 43 patients (37.4%) had three systemic diseases. Postoperative pain was assessed one hour after surgery using VAS and yielded a median score of 4 ± 1 (range 0-10). The mean hospital stay was 12 hours, and the mean postoperative catheterization duration was three days.

In the early postoperative period (within 30 days), urinary retention occurred in seven patients (6.08%), urinary tract infection in four patients (3.4%), and transient urinary incontinence (lasting up to 15 days) in two patients (1.73%). Patients who developed postoperative urinary retention required re-catheterization for 72 hours, after which all were able to void spontaneously. Perioperative complications were graded according to the Clavien-Dindo classification. No complications requiring blood transfusion were encountered, nor were there cases of sepsis, bladder perforation, or prostatic capsule perforation. No grade IV or V complications were observed.

Comparison of preoperative parameters with those at postoperative month 3 showed a significant reduction in prostate volume (mean change −7.77 mL, 95% CI: −9.4 to −6.1, p = 0.001), significant improvements in IPSS (mean change −3.82, 95% CI: −4.5 to −3.1, p = 0.001) and QoL (mean change −1.93, 95% CI: −2.4 to −1.4, p = 0.01), a significant increase in Qmax (mean change +2.65 mL/s, 95% CI: 2.1-3.2, p = 0.001), and a significant decrease in PVR (mean change −29.7 mL, 95% CI: −38.2 to −21.3, p = 0.001). However, no significant change was observed in PSA levels (p = 0.976) (Table 2).

**Table 2:** Pre‐ and post-operative (months 3 and 12) comparisons. Descriptive statistics are presented as mean ± standard deviation; * values marked with an asterisk () are statistically significant (p < 0.05); lettering (ᵃ, ᵇ, ᶜ) is applied row-wise; there is no difference between identical letters. Qmax: Maximum flow rate of urination; PVR: Post-void residual volume; IPSS: International Prostate Symptom Score; QoL: Quality of Life.

Variable	Preoperative	Postoperative month 3	Postoperative month 12	p-value
Qmax (ml/s)	10.30 ± 3.76ᵃ	12.95 ± 4.03ᵇ	14.39 ± 4.50ᶜ	0.001*
PVR (ml)	137.56 ± 104.48ᵃ	107.82 ± 77.35ᵇ	101.82 ± 83.31ᵇ	0.001*
IPSS	19.54 ± 6.16ᵃ	15.72 ± 5.42ᵇ	13.79 ± 4.48ᶜ	0.001*
Prostate volume (cc)	57.68 ± 29.41ᵃ	49.91 ± 25.47ᵇ	48.25 ± 23.89ᶜ	0.001*
PSA (ng/ml)	7.55 ± 26.87	8.33 ± 40.11	8.01 ± 34.45	0.976
QoL	4.55 ± 1.17	2.62 ± 1.59	2.67 ± 1.67	0.010*

At postoperative month 12, prostate volume remained significantly decreased (mean change −9.43 mL, 95% CI: −11.2 to −7.6, p = 0.001), Qmax significantly increased (mean change +4.09 mL/s, 95% CI: 3.2-4.9, p = 0.001), IPSS significantly improved (mean change −5.75, 95% CI: −6.5 to −5.0, p = 0.001), PVR significantly decreased (mean change −35.7 mL, 95% CI: −45.8 to −25.6, p = 0.001), and QoL showed significant improvement (mean change −1.88, 95% CI: −2.3 to −1.4, p = 0.001) compared to preoperative values (Table 2).

**Figure 1:**
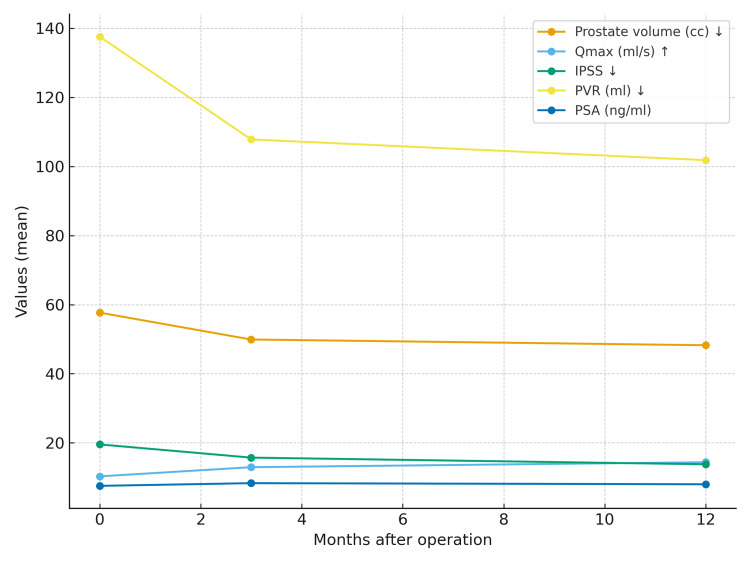
Trajectory of postoperative data in the first 12 months. There was a more rapid reduction in prostate volume, a more rapid increase in Qmax, a more pronounced improvement in IPSS, and a faster decline in PVR in the first three months. The rate of change in these values was found to be decreased after the third month. PSA levels increased during the first three months, followed by a decline. Qmax: Maximum flow rate of urination; PVR: Post-void residual volume; IPSS: International Prostate Symptom Score; QoL: Quality of Life; PSA: Prostate-specific antigen.

During the first 12 months, prostate volume reduction, improvement in IPSS, increase in Qmax, and decline in PVR were most rapid during the first three months, with a slower rate of change thereafter. In contrast, PSA levels initially increased in the first three months, followed by a subsequent decline (Figure 1).

Subgroup analysis comparing patients with prostate volumes <80 mL versus ≥80 mL at postoperative months 3 and 12 demonstrated that surgical outcomes were significantly superior in prostates <80 mL (p < 0.05). At both time points, patients with smaller prostates showed greater improvements in PVR (mean difference −62.9 mL, 95% CI: −84.2 to −41.6, p = 0.001), IPSS (mean difference −3.9, 95% CI: −5.4 to −2.4, p = 0.002), and QoL (mean difference −1.8, 95% CI: −2.4 to −1.2, p = 0.010). The only exception was Qmax, where no statistically significant difference was observed between the groups (p > 0.05) (Table 3).

**Table 3:** Comparison of three-month and 12-month outcomes for prostates smaller and larger than 80 cc. Numerical variables are reported as mean ± standard deviation; ‡: Independent Samples t-test; †: Mann–Whitney U test. PV: Prostate volume; Qmax: Maximum flow rate of urination; PVR: Post-void residual volume; IPSS: International Prostate Symptom Score; QoL: Quality of Life; PSA: Prostate-specific antigen.

Variable	PV < 80 cc	PV > 80 cc	p-value
Postoperative month 3			
Qmax (ml/s)	13.16 ± 3.98	11.34 ± 4.31	0.164†
PVR (ml)	100.65 ± 74.9	163.54 ± 76.16	0.001†
IPSS	15.31 ± 5.11	18.92 ± 6.82	0.041†
Prostate volume (cc)	43.03 ± 16.57	103.38 ± 18.04	0.001†
QoL	1.33 ± 0.68	4.06 ± 1.04	0.010
PSA (ng/ml)	8.66 ± 42.6	5.82 ± 4.37	0.002†
Postoperative month 12			
Qmax (ml/s)	14.63 ± 4.45	11.97 ± 4.52	0.122†
PVR (ml)	94 ± 75.26	183.2 ± 119.36	0.010†
IPSS	13.32 ± 4.19	18.7 ± 4.62	0.002†
Prostate volume (cc)	42.98 ± 17.03	103 ± 14.08	0.001†
QoL	1.29 ± 0.63	4.00 ± 1.07	0.010
PSA (ng/ml)	8.27 ± 36.04	5.33 ± 5.28	0.008†

## Discussion

Lower urinary tract symptoms (LUTS) are highly prevalent in aging males [1,2]. In older men, the most frequent cause of LUTS is benign prostatic obstruction (BPO) [3,4]. Approximately 37% of patients treated medically for BPO eventually require surgical intervention with catheterization [6,7]. Since most of these patients are older than 65 years and frequently have comorbidities such as coronary heart disease, hypertension, or diabetes mellitus, surgical procedures requiring general or spinal anesthesia (e.g., TURP or holmium laser enucleation of the prostate (HoLEP)) are often associated with increased risk. In contrast, transurethral microwave thermotherapy of the prostate (TEMPRO) can be performed under local anesthesia and does not require interruption of anticoagulation therapy.

In this study, the median patient age was 73.7 ± 11.2 years. This was higher than that reported by Diri and Gul (63.2 ± 6.7 years) [14], but comparable to Salar et al. (72 years) [15]. Advanced age is generally associated with multiple comorbidities, which may negatively influence surgical outcomes.

A significant reduction in prostate volume was observed at 3 and 12 months, with the greatest change within the first three months. Previous reports did not consistently evaluate this outcome: Diri and Gul did not include pre- and postoperative prostate size [14]; Benli et al. found no significant changes up to six months [16]. Therefore, our findings suggest that TEMPRO may contribute to prostate shrinkage, which could be clinically relevant.

Significant improvements in IPSS and quality of life (QoL) were found at both 3 and 12 months. These results are consistent with earlier studies: Diri and Gul reported improvement at six months [14], Salar et al. described persistent improvement up to 24 months [15], and Benli et al. noted significant benefit by six months [16]. Collectively, these findings highlight the efficacy of TEMPRO in reducing LUTS and improving QoL.

TEMPRO was also effective in improving maximum urinary flow rate (Qmax) and post-void residual (PVR) at 3 and 12 months, in agreement with other reports [14-16]. However, although Qmax improved significantly, median values generally remained below 15 ml/s, indicating that flow restoration to near-normal levels may be limited.

No significant reduction in PSA was noted within 12 months. Instead, PSA values increased during the first three months, likely due to necrosis rather than resection of tissue, a pattern resembling PSA behavior after radiotherapy in localized prostate cancer. Salar et al. reported PSA reduction at 24 months [15], suggesting that longer follow-up may reveal different trends.

Our findings showed greater treatment success in prostates <80 mL, which is in line with Diri and Gul, who reported better outcomes in prostates <70 mL [14]. Prostate size thus appears to be an important factor in predicting clinical response.

Urinary retention developed in seven patients, all of whom were managed with temporary catheterization and regained spontaneous voiding within 72 hours. In Salar et al.’s series, 27% of patients experienced retention [15], likely influenced by prolonged preoperative catheterization leading to detrusor dysfunction. Importantly, no cases of sepsis, transfusion-requiring bleeding, or Clavien grade IV-V complications occurred, confirming the favorable safety profile of TEMPRO.

Pain levels were higher in our study (VAS 4 ± 1 at postoperative hour 1) compared to Diri and Gul (VAS 1 at postoperative hour 6) [14]. This discrepancy likely reflects differences in the timing of assessment.

This study has several limitations. The main limitation is its relatively short follow-up period compared with previous reports. In addition, the sample size was relatively small, which may limit the generalizability of the findings. Furthermore, the retrospective design of the study may introduce potential selection bias and restrict the ability to establish causal relationships. Longer-term prospective studies with larger cohorts are needed to more accurately evaluate PSA dynamics and the durability of clinical outcomes.

## Conclusions

Within the limits of this retrospective study, TEMPRO appears to be a safe and potentially effective treatment for benign prostatic obstruction in elderly patients who are not suitable for spinal or general anesthesia due to comorbidities. Its minimally invasive nature makes it particularly valuable for frail populations with a high anesthetic risk.

## References

[1] Chapple C, Abrams P (2013). Male Lower Urinary Tract Symptoms (LUTS): An International Consultation on Male LUTS. http://www.siu-urology.org/themes/web/assets/files/ICUD/pdf/Male%20Lower%20Urinary%20Tract%20Symptoms%20%28LUTS%29.pdf.

[2] Martin SA, Haren MT, Marshall VR, Lange K, Wittert GA (2011). Prevalence and factors associated with uncomplicated storage and voiding lower urinary tract symptoms in community-dwelling Australian men. World J Urol.

[3] Abrams P, Cardozo L, Fall M (2002). The standardisation of terminology of lower urinary tract function: report from the Standardisation Sub-committee of the International Continence Society. Neurourol Urodyn.

[4] Kupelian V, Wei JT, O'Leary MP, Kusek JW, Litman HJ, Link CL, McKinlay JB (2006). Prevalence of lower urinary tract symptoms and effect on quality of life in a racially and ethnically diverse random sample: the Boston Area Community Health (BACH) Survey. Arch Intern Med.

[5] Foster HE, Barry MJ, Dahm P (2018). Surgical management of lower urinary tract symptoms attributed to benign prostatic hyperplasia: AUA guideline. J Urol.

[6] Millán-Rodríguez F, Chéchile-Toniolo G, Palou-Redorta J, Ponce de Leon X, Salvador-Bayarri J (1999). Re: 5-year outcome of surgical resection and watchful waiting for men with moderately symptomatic benign prostatic hyperplasia: a Department of Veterans Affairs cooperative study. J Urol.

[7] Wasson JH, Reda DJ, Bruskewitz RC, Elinson J, Keller AM, Henderson WG (1995). A comparison of transurethral surgery with watchful waiting for moderate symptoms of benign prostatic hyperplasia. The Veterans Affairs Cooperative Study Group on transurethral resection of the prostate. N Engl J Med.

[8] Cornu JN, Ahyai S, Bachmann A (2015). A systematic review and meta-analysis of functional outcomes and complications following transurethral procedures for lower urinary tract symptoms resulting from benign prostatic obstruction: an update. Eur Urol.

[9] Madersbacher S, Lackner J, Brössner C, Röhlich M, Stancik I, Willinger M, Schatzl G (2005). Reoperation, myocardial infarction and mortality after transurethral and open prostatectomy: a nation-wide, long-term analysis of 23,123 cases. Eur Urol.

[10] Eredics K, Wachabauer D, Röthlin F, Madersbacher S, Schauer I (2018). Reoperation rates and mortality after transurethral and open prostatectomy in a long-term nationwide analysis: have we improved over a decade?. Urology.

[11] Allen S, Aghajanyan I (2017). Use of thermobalancing therapy in ageing male with benign prostatic hyperplasia with a focus on etiology and pathophysiology. Aging Male.

[12] Devonec M, Ogden C, Perrin P, St Clair Carter S (1993). Clinical response to transurethral microwave thermotherapy is thermal dose dependent. Eur Urol.

[13] Dindo D, Demartines N, Clavien PA (2004). Classification of surgical complications: a new proposal with evaluation in a cohort of 6336 patients and results of a survey. Ann Surg.

[14] Diri MA, Gul M (2020). Effect of bipolar radiofrequency thermotherapy on benign prostate hyperplasia. Andrologia.

[15] Salar R, Özbay E, Öncel HF (2021). Bipolar radiofrequency thermotherapy treatment of the prostate in urinary catheter-dependent men. Low Urin Tract Symptoms.

[16] Benli E, Yuce A, Nalbant I, Cirakoglu A, Yazici I (2020). Can transurethral thermotherapy save elderly patients with benign prostatic obstruction and high ASA score?. Aging Male.

